# The Potential Use of Near- and Mid-Infrared Spectroscopy in Kidney Diseases

**DOI:** 10.3390/ijms24076740

**Published:** 2023-04-04

**Authors:** Charlotte Delrue, Sander De Bruyne, Marijn M. Speeckaert

**Affiliations:** 1 Department of Nephrology, Ghent University Hospital, 9000 Ghent, Belgium; 2 Department of Diagnostic Sciences, Ghent University Hospital, 9000 Ghent, Belgium; 3 Research Foundation-Flanders (FWO), 1000 Brussels, Belgium

**Keywords:** infrared spectroscopy, mid-infrared, near-infrared, attenuated total reflectance-Fourier-transform infrared spectroscopy, kidney diseases

## Abstract

Traditional renal biomarkers such as serum creatinine and albuminuria/proteinuria are rather insensitive since they change later in the course of the disease. In order to determine the extent and type of kidney injury, as well as to administer the proper therapy and enhance patient management, new techniques for the detection of deterioration of the kidney function are urgently needed. Infrared spectroscopy is a label-free and non-destructive technique having the potential to be a vital tool for quick and inexpensive routine clinical diagnosis of kidney disorders. The aim of this review is to provide an overview of near- and mid-infrared spectroscopy applications in patients with acute kidney injury and chronic kidney disease (e.g., diabetic nephropathy and glomerulonephritis).

## 1. Introduction

Acute kidney injury (AKI) and chronic kidney disease (CKD) are caused by several medical conditions [1]. In clinical practice, the glomerular filtration rate (GFR) and albuminuria/proteinuria are most frequently used to identify kidney disorders. More specifically, serum creatinine has been the gold standard for nearly a century, despite the shortcomings of this biomarker [2,3]. First, serum creatinine concentrations are not specific to the etiology of kidney disease. They can be influenced by other non-renal variables such as age, sex, and body weight. Second, serum creatinine reflects not only renal excretion, but also the generation, intake, and metabolism of creatinine. It is a rather insensitive biomarker since it changes later in the course of the disease when there is a significant reduction in the glomerular filtration rate. In a healthy individual, nearly 50% of the kidney function must be lost before an increase in serum creatinine is detectable. This is because the kidneys are able to compensate for lost function by increasing the activity of the remaining nephrons (the functional units of the kidney) to maintain overall filtration [4,5,6].

It is imperative to develop novel methods for the rapid and precise identification of biochemical changes in biological samples associated with specific medical phenotypes, such as kidney disease. Nuclear magnetic resonance (NMR) spectroscopy and a variety of mass spectrometry (MS) techniques provide good selectivity and specificity for the identification of specific metabolites, which can be analyzed in the future using more straightforward techniques, such as biosensors. Hope is raised for significant cost reductions and potentially ground-breaking improvements in diagnosis thanks to the prospective application of vibrational spectroscopy techniques [7]. Vibrational spectroscopy, including infrared (IR) and Raman (RS) spectroscopy, can precisely measure a polyatomic molecule’s vibrational spectra based on linear absorption and inelastic light scattering techniques, respectively. The chemical composition of the molecule, its interaction with its surroundings, and dynamic transitions between various conformations have a significant impact on these spectra. Therefore, vibrational spectroscopy has become a vital technique in the investigation of functional materials, as well as in chemical and biological experiments [7,8,9,10,11,12,13,14,15,16,17,18].

**Figure 1:**
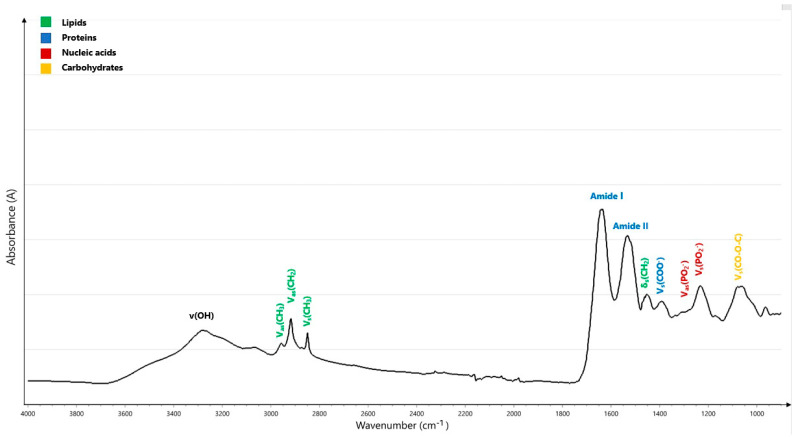
Typical MIR spectrum measured using attenuated total reflection–Fourier transform infrared (ATR–FTIR) spectroscopy of a biological sample showing peak assignments from 4000 to 800 cm^−1^. V: stretching vibrations, δ: bending vibrations, s: symmetric vibrations, as: asymmetric vibrations.

The IR spectral region, ranging between 0.80 µm and 1000 µm, can be further subdivided into the near-infrared (NIR: 0.8 µm to 2.5 µm), mid-infrared (MIR: 2.5 µm to 25 µm), and far-infrared bands (FIR: 25–1000 µm) [19]. The MIR region consists of four regions: the X-H stretching region (4000–2500 cm^−1^), the triple-bond region (2500–2000 cm^−1^), the double-bond region (2000–1500 cm^−1^), and the fingerprint region (1500–1600 cm^−1^). The fingerprint region and the double-bond region, which indicate four conformationally sensitive protein bands, amide I, II, III, and A vibrations, are the most crucial regions for the analysis of biological materials [20]. The amide I region (~1650 cm^−1^) is primarily caused by the vibration of C double-bond stretching (C=O). The amide II band (~1550 cm^−1^) is related to C-N stretching and N-H in-plane bending. The amide III vibration (1400–1200 cm^−1^) is characterized by C-N stretching, N-H in-plane bending, and C=O in-plane bending, and, finally, amide A vibration is caused by N-H stretching *(*Table 1) [4,5,18,21,22]. Numerous molecular interactions that create complicated patterns in the fingerprint region can be utilized to identify various substances. Because of this complexity and the presence of many overlapping bands, the generated spectrum must be analyzed using mathematical and statistical methods [4]. A biological MIR spectrum with normal molecular assignments is displayed in Figure 1.

Three regions can be identified in the NIR spectrum: region I (13,500–8500 cm^−1^), region II (8500–5500 cm^−1^), and region III (5500–4000 cm^−1^) [18,23]. Since the spectral range is shorter than the MIR range, molar absorptivity in the NIR region is normally relatively low, but the sample penetration depth is increased [18,24,25]. MIR radiation penetrates connective tissue at a depth of only 10 microns, whereas NIR penetrates human cartilage at depths of up to 5 mm [26]. The two mechanisms that produce the major absorptions in the NIR region are combinations of the basic vibrations of the -CH, -NH, -OH (and -SH) functional groups, and overtones [27]. Although there are only a few potential overtones from a set of absorptions in a molecule, a relatively wide number of combinations are observed [28]. The NIR spectrum can be measured for every hydrogen-containing molecule [18,29,30]. In NIR spectral analysis, light is attenuated by three factors: (1) chromophores with fixed concentrations (melanin, water, collagen, and lipids); (2) oxygen (O_2_)-dependent chromophores with variable concentrations (oxyhemoglobin, deoxyhemoglobin, cytochrome c oxidase, and myoglobin); and (3) light scattering [31]. Differential absorption spectra of oxygen-dependent chromophores in the NIR range enable spectroscopic separation as well as measurements of tissue oxygenation and blood flow [27]. Although both the NIR and MIR are components of the IR spectrum, most researchers have focused on the MIR part of the spectrum. Since the MIR region encompasses fundamental vibration bands related to the functional groups in the sample, this method provides sharper bands and more information on disease diagnosis than the overtone and harmonic vibrations that are provided by the NIR region [8].

The recent literature lacks comprehensive reviews that aim to illustrate the current state and the future potential of NIR and MIR spectroscopy in patients with kidney disease. Therefore, this review aims to briefly summarize the potential utility of both methods in AKI and CKD (diabetic nephropathy (DN) and glomerulonephritis (GN)).

## 2. Methodology

NIR spectra can be collected using a dedicated NIR spectrometer, UV-visible-NIR absorption spectrometer, or Fourier-transform infrared (FTIR) spectrometer with an NIR extension. The most significant advantage of NIR spectroscopy is the intact sampling handling. Additionally, NIR spectroscopy has three measurement modes: reflection, transflection, and transmission. The instrumentation for NIR spectroscopy is tough, flexible, and even portable, and can be used in a process environment. NIR spectra contain a wealth of information about the chemical and physical properties of a sample, which must be extracted with considerable effort. Chemometric data analysis algorithms are used because the NIR spectrum is composed of overlapping overtones and combinations of bands originating in the mid-IR spectrum. Despite the fact that multiple-component quantitation is now routinely performed, NIR spectroscopy is not without development issues. Each application requires a relatively large number of samples for accurate calibration, as well as the development and maintenance of a calibration routine. NIR spectroscopy is not a technique for analyzing components other than those in the training set. NIR spectroscopy is a non-destructive, cost-effective, and user-friendly process tool that allows for simultaneous qualitative and quantitative analysis of multiple parameters, high sample throughput, and real-time monitoring. However, it is not a method for determining trace amounts. For liquid media analyses, the detection limits are in the low percentage range, and in some cases in the high per mil range. NIR spectroscopy is used to study samples, which are often very complex mixtures. Because FT-NIR spectroscopy requires only one instrument for multiple measurements, the testing protocol for process samples is simplified, multiple pieces of equipment can be replaced, and laboratory supply costs are reduced. Furthermore, the ability to analyze samples for multiple components much faster (30 s) than traditional primary analytical laboratory methods (up to 1 h) results in significant time savings. However, several limitations have also been associated with NIR spectroscopy, such as the need for a secondary method (calibration against the reference method), dependence on a large reference set, the influence of sample morphology, slow and costly method development, the need for a quantitative calibration model, troublesome calibration transfer, strict sample temperature control, spectroscopic complexity, a lack of a structural interpretative value, a lack of reference data, the need for sophisticated data evaluation algorithms, and weak sensitivity to minor components [32].

The application of MIR spectra has been increased by the use of the Fourier transform algorithm, several chemometric tools, and different available sampling methods such as transmission, transflection, and attenuated total reflectance (ATR) [33]. FTIR spectroscopy is a label-free and non-destructive technique [4,16,17,20]. This technique simultaneously directs a beam of light at various frequencies to a sample [4,5]. When a molecule interacts with IR light, the chemical bonds vibrate more vigorously, changing the vibration and rotation of the molecule. However, only a few energy levels are permitted because of the limitations imposed on atoms by quantum physics [18]. A charge-coupled device (CCD) measures the amount of laser beam absorbed by the samples. A second data point is then obtained by altering the beam to comprise a mix of different frequencies. Using a Fourier transform algorithm, a computer uses all of these data to determine the exact absorption of the sample at each wavelength, resulting in the chemical fingerprint of biomolecules [4,5]. This chemical fingerprint relies on the interaction between the chemical or biochemical substances in the sample and the IR beam, which is absorbed by the functional groups in the sample and vibrates as a result of stretching (sole vibration if there are only two atoms), bending, deformation, or a combination [8,18]. Multiplex (or Fellgett’s), throughput (or Jacquinot’s), and registration (or Connes’) are three major practical advantages of FT spectroscopy, according to the literature [34]. The SNR associated with detector characteristics improves with multiplex gain. In contrast to dispersive measurement, an interferogram records superimposed signals from all wavelengths, and each wavelength is presented to the detector for an N-times longer period, resulting in a single-to-noise ratio (SNR) advantage of √N [35]. The throughput advantage is due to the optics; a circular aperture in an FT spectrometer passes light more effectively than a slit in dispersive spectrometers [36]. These two gains improve the SNR, which is further improved by the more effective averaging of multiple scans, which can be collected in much less time in FT spectrometry [37]. One of the most difficult challenges in the field is reaching an agreement on spectral pre-processing and data analysis [17]. Pre-processing aims primarily to improve the robustness and accuracy of subsequent multivariate analyses, as well as to improve data interpretability by correcting issues associated with spectral data acquisition [38]. De-noising, spectral correction, normalization, and other manipulations are examples of pre-processing methods. Two or three methods are frequently used for this purpose. The pre-processing methods used may be determined by the analysis goal, the physical state of the sample, and the amount of time and computing power available [34]. IR spectra can be de-noised using Savitzky–Golay (SG) smoothing, minimum noise fraction [39], or wavelet de-noising (WDN) [40]. Another option is to use principal component analysis (PCA) to decompose the spectra and then reconstruct them using only a few of their principal components (PCs), thereby discarding those PCs that are mostly noisy [41,42]. Multivariate data analysis elucidates potential diagnostic markers, resulting in a fast and label-free technology that can be used alongside traditional techniques, such as histology [15,43].

## 3. Kidney Diseases

### 3.1. Acute Kidney Injury after Cardiac Surgery

Cardiac-surgery-associated acute kidney injury (CSA-AKI) is the most common complication of heart surgery in both pediatric and adult patients, with an incidence ranging between 5 and 40%. It is also associated with increased in-hospital mortality and morbidity. Regardless of other risk factors and even in individuals who have fully recovered kidney function, the risk of death associated with AKI remains significant for 10 years after cardiac surgery [28,44,45,46,47,48]. There is currently no consensus in the literature on the constituents of CSA-AKI. According to various diagnostic methodologies, over 35 alternative definitions of CSA-AKI have been reported [28,49]. The majority of researchers have utilized the kidney disease: improving global outcomes (KDIGO), the (pediatric) risk, injury, failure, loss, end-stage kidney disease ((p)RIFLE), or acute kidney injury network (AKIN) criteria, which all rely on changes in serum creatinine and urine output [28,46,48,49,50,51]. Although these measures may change in response to kidney damage, they are rather late and insensitive AKI indicators. Additionally, the validity of the pRIFLE and AKIN criteria following newborn heart surgery is unknown [46]. For adults, preoperative risk factors for the occurrence of CSA-AKI frequently include female sex, advanced age, and the presence of numerous comorbidities (such as pre-existing CKD, prior cardiac surgery, obesity, chronic obstructive pulmonary disease, diabetes mellitus, hypertension, hypercholesterolemia, congestive heart failure, and left ventricular ejection fraction <35%) [28]. In children, young age, low weight, prolonged cardiopulmonary bypass (CPB), and postoperative hemodynamic instability are well-documented risk factors [52,53,54]. In order to identify more precisely those who are at risk and enable earlier detection and intervention, novel biomarkers of kidney injury have been developed: neutrophil gelatinase-associated lipocalin (NGAL), cystatin C, L-type fatty acid binding protein (L-FABP), interleukin-8 (IL-8), insulin-like growth-factor-binding protein 7 (IGFBP-7), and tissue inhibitor of metalloproteinases 2 (TIMP 2) [46,55,56,57]. However, none of these tests can be performed in real-time [50,55].

Because AKI is common and may have long-term consequences that are unfavorable after cardiac surgery, reducing AKI is of utmost importance. Kidney injury following cardiac surgery may be undetectable by functional assessment alone (creatinine level), and the continuous monitoring of renal regional tissue oximetry may be more sensitive to significant subclinical AKI. Renal rSO_2_ monitoring is non-invasive and offers continuous real-time data on the equilibrium between tissue oxygen supply and demand. Currently, NIR is used to track cerebral rSO_2_ during cardiac surgery. Poor neurologic outcomes, including stroke and postoperative cognitive dysfunction, are linked to intra-operative cerebral oxygen desaturation [58,59,60]. However, NIR technology is not used during cardiac surgery for the monitoring of kidney function in clinical practice, yet it could serve as a possible solution for the earlier detection of CSA-AKI [45,46,61]. In multiple studies [50,55,58,61,62,63], CSA-AKI has been linked to lower renal rSO_2_ values both intra-operatively (i.o.) and during the first 48 h postoperatively (p.o.) (Table 2). Two different wavelengths of NIR light (730 and 810 nm) correspond to the spectral absorptions of oxygenated and deoxygenated hemoglobin, respectively [47]. As an example, a prospective cohort study investigated 59 infants undergoing cardiac surgery for congenital heart disease (CHD). AKI patients had significantly lower renal rSO_2_ values, which continued during the first 12, 24, and 48 h p.o., compared to patients with normal kidney function (*p* < 0.05 for all time-points) [61]. However, in another study [61], p.o. rather than i.o. rSO_2_ reduction was associated with the development of CSA-AKI. These results emphasize the necessity of recording rSO_2_ after cardiac surgery, because continuous monitoring appears to be insufficient. Many factors affect the development of CSA-AKI even after surgery. These include low cardiac output syndrome, systemic inflammatory response, adrenergic discharge, vasoplegia, anemia, excessive bleeding, sepsis, and nephrotoxins (antibiotics, contrast media, angiotensin-converting enzyme inhibitors, cephalosporins, aminoglycosides, and nonsteroidal anti-inflammatory drugs) [62]. Therefore, it makes sense to regularly monitor renal rSO_2_, even after surgery, to enable the best management of the kidney state.

Several cut-off values of rSO_2_ have been investigated for the optimal identification of patients at risk of CSA-AKI. A prospective cohort study assessed renal oxygenation in 50 neonates and infants who underwent repair (*n* = 31) or neonatal palliation (*n* = 19) for CHD. For a cut-off value of 64.8%, the sensitivity of rSO_2_ in predicting AKI was 78–80% and the specificity was 63–65% with an area under the curve (AUC) of 0.68–0.83 [63]. The receiver operating characteristic (ROC)-AUC analysis confirmed a valuable diagnostic accuracy for renal rSO_2_ < 65% at baseline (AUC: 0.689; 95% CI: 0.573–0.785; sensitivity = 71.4%; specificity = 67.4%; *p* = 0.002), but provided evidence for the diagnostic accuracy of a decrease in renal rSO_2_ > 20% p.o. with a sensitivity of 67.6% and specificity of 51.9% (AUC: 0.639; 95% CI: 0.523–0.755; *p* = 0.019) [62]. A prospective cohort study of 242 infants (1–12 months) undergoing ventricular septum defect (VSD) repair involving CPB showed that infants with renal desaturation (defined as an rSO_2_ decrease of ≥20% from the baseline level for at least 60 consecutive seconds) had significantly higher odds of developing CSA-AKI in the first three days p.o. (odds ratio (OR): 2.79, 95% confidence interval (CI): 1.21–6.44, *p* = 0.016). Additionally, renal desaturation was associated with a 2-fold increased risk of CSA-AKI (hazard ratio (HR) = 2.06, 95% CI: 1.14–3.74, *p* = 0.017) [64]. As mentioned above, urinary NGAL levels may be a good biomarker for kidney function showing a negative correlation with rSO_2_ [45,50]. At baseline, 2 h, 12 h, and 24 h p.o., there were significant inverse associations between i.o. renal rSO_2_ levels and NGAL (r = −0.356, *p* = 0.014; r = −0.332, *p* = 0.034; r = −0.33, *p* = 0.017; and r = −0.343, *p* = 0.01, respectively). In fact, significantly increased NGAL at all p.o. time-points was linked with an i.o. renal rSO_2_ drop of ≥30% from baseline, indicating that newborns and babies with sustained higher renal oxygenation during surgery might experience less perioperative kidney injury [50]. However, not all studies confirmed this correlation [61]. A prospective cohort study [62] of 87 children found that renal rSO_2_ values < 65%, 60%, and 55% were significantly related to CSA-AKI (*p* = 0.005, *p* = 0.001, *p* = 0.001, and *p* = 0.013, respectively). In addition, declines in renal rSO_2_ > 15%, 20%, 25%, and 30% from baseline were (borderline) significantly related to p.o. CSA-AKI (*p* = 0.05, *p* = 0.013, *p* < 0.001, and *p* = 0.009, respectively). More specifically, a decrease in renal rSO_2_ ≥ 25% during CPB was independently associated with the development of CSA-AKI (OR: 3.90; 95% CI: 1.68–9.02; *p* = 0.002). Similar findings have been reported for adults. A prospective cohort study [58] of 95 adult patients undergoing elective cardiac surgery under CPB showed that the period of renal desaturation < 65% and the number of patients with a decrease in renal rSO_2_ > 20% were significantly higher in AKI patients than in patients with normal kidney function (*p* = 0.001 and *p* = 0.030, respectively). AUC-ROC analysis for renal rSO_2_ values < 55% for >1.5 min showed a sensitivity of 64.7% and a specificity of 88.5% (AUC = 0.777, 95% CI: 0.669–0.885; *p* < 0.001) for predicting CSA-AKI. Nonetheless, the usefulness of renal rSO_2_ measurements in detecting CSA-AKI could not be confirmed in other investigations [65,66], and two studies even suggested that low renal rSO_2_ values before and during CPB may have kidney-protective effects [47,66]. In a small prospective cohort study of 66 infants (≤10 kg) undergoing CHD repair with CPB, infants with lower baseline and i.o. renal rSO_2_ values had lower odds of developing CSA-AKI (OR: 0.95, 95% CI: 0.91–0.99, *p* = 0.01; OR = 1.06, 95%CI: 1.01–1.12, *p* = 0.02, respectively) [47]. These disparities might be explained by (1) different study population groups, (2) lower rates of severe AKI, (3) a more significant and prolonged decline in renal NIRS, and (4) a different cause of AKI (i.e., nephrotoxic AKI might not be associated with a decline in renal NIRS) [66].

In addition to the value of renal rSO_2_ measurements, some studies have investigated the use of intestinal, cerebral, and thigh rSO_2_ quantification [55,62,67]. A prospective cohort study of 57 children who underwent CHD surgery investigated the added value of intestinal and cerebral rSO_2_. Both renal and intestinal rSO_2_ values were significantly lower in AKI patients at 48 h p.o. (both *p* < 0.01) [55]. However, no significant decrease in cerebral rSO_2_ was observed in patients with AKI [55,62]. Intestinal rSO_2_ has the potential to be used as an index to predict AKI within 24 h following surgery. AUC analysis revealed a good diagnostic accuracy of intestinal and renal rSO2 p.o. for AKI ≥ grade 2, with cut-off values of 84% at 3 h p.o. and 71% at 31 h p.o., respectively (AUC: 0.826; sensitivity = 91.9%; specificity = 55%; AUC: 0.829; sensitivity = 91.9%; specificity = 55%, respectively). More specifically, these results indicate that renal rSO_2_ has the best predictive performance 24–48 h after surgery, but intestinal rSO_2_ has the potential to be utilized as an index to predict AKI within 24 h following surgery. For thigh rSO_2_ values, a significant positive correlation with eGFR was found in a prospective cohort study in 150 adults, not on dialysis, who underwent elective cardiac surgical procedures (r = 0.31, 95% CI: 0.16–0.45, *p* < 0.001) [55].

More research is needed to determine how to use rSO_2_ monitoring to successfully detect renal ischemia and hypoxia and to provide an early warning. However, the definition of pathological rSO_2_ readings varies across studies and does not consider the severity and length of desaturation. These need to be further specified in future investigations to introduce renal rSO_2_ into standard clinical practice [61]. Investigating how to successfully reverse a negative decline in renal rSO_2_ and whether rSO_2_-guided therapy lowers AKI are crucial next steps. Randomized controlled trials are required to answer these questions [64].

**Table 2:** Overview of near-infrared and mid-infrared spectroscopy studies in patients with acute kidney injury after cardiac surgery, in preterm infants with acute kidney injury, and in patients with chronic kidney disease, diabetic nephropathy, and glomerulonephritis. AKI: acute kidney injury; ATR-FTIR: attenuated total reflectance-Fourier-transform infrared; AUC: area under the curve; CABG: coronary artery bypass grafting; CHD: congenital heart disease; CI: confidence interval; CKD: chronic kidney disease; CPB: cardiopulmonary bypass; CS: cardiac surgery; CSA-AKI: cardiac-surgery-associated acute kidney injury; DN: diabetic nephropathy; (e)GFR: (estimated) glomerular filtration rate; GN: glomerulonephritis; HCs: healthy controls; HbA1c: hemoglobin A1c; HR: hazard ratio; IGFBP7: insulin-like growth-factor-binding protein 7; IL-18: interleukin-18; i.o.: intra-operatively; IR: infrared; KDIGO: kidney disease: improving global outcomes; MIR: mid-infrared; NIR(S): near-infrared (spectroscopy); NL: normal kidneys; NLD: normal kidneys of diabetic individuals; rSO_2_: regional oxygen saturation; TIMP2: tissue inhibitor of metalloproteinases 2; VSD: ventricle septum defect.

Pathology	Spectral Region	Study Design	Study Population	Major Findings	Ref
AKI after cardiac surgery	NIR	Prospective cohort study	49 infants (≤6 months) undergoing CHD repair with CPB	Infants at high risk of poor outcomes had elevated urinary NGAL, IL-18, and cystatin C levels, as well as more time with low NIRS saturation (rSO_2_ < 50%) within the first 24 h after CPB.	[45]
	NIR	Prospective cohort study	40 infants (<12 months) undergoing biventricular repair	In infants with CHD undergoing biventricular repair, prolonged low renal NIR oximetry appeared to correlate with kidney dysfunction, decreased systemic oxygen delivery, and an overall postoperative course.	[46]
	NIR	Prospective cohort study	66 children (≤10 kg) undergoing CHD repair with CPB	A lower baseline rSO_2_ was associated with a lower risk of CSA-AKI (*p* = 0.01). Children with the highest tertial baseline rSO_2_ were 7.14 times more likely to develop CSA-AKI (vs. lowest tertile). The AUC for baseline rSO_2′_s ability to predict CSA-AKI was 0.73 (95% CI: 0.60–0.85). The mean renal rSO_2_ was lower in children with a lower baseline GFR.	[47]
	NIR	Prospective cohort study	70 infants (≤12 months) undergoing CHD repair with CPB	There were significant correlations between renal rSO_2_, TIMP2, IGFBP7, and postoperative NGAL levels in AKI patients.	[50]
	NIR	Prospective cohort study	57 children undergoing CHD surgery (weight >2.5 kg and age ≤1 year)	Monitoring intestinal and renal rSO_2_ levels after surgery can predict the occurrence and severity of postoperative AKI in children with CHD.	[55]
	NIR	Prospective cohort study	95 adult patients (mean age ± 60 years) undergoing CS under CPB	Intra-operative renal rSO_2_ may be a good predictor of AKI in adult patients undergoing surgery.	[58]
	NIR	Prospective cohort study	59 infants undergoing CHD repair with CPB	Long-term low-renal rSO_2_ during CS was associated with AKI development and may be superior to traditional biochemical markers. Renal NIRS could be a promising non-invasive multimodal monitoring tool for kidney function and the development of AKI in infants undergoing CS with CPB.	[61]
	NIR	Prospective cohort study	121 adults (median age: 70 years) undergoing CS under CPB	CSA-AKI development has been linked to postoperative kidney oxygen saturation. Continuous renal rSO_2_ monitoring might be a promising non-invasive tool for AKI in adult patients following CS.	[62]
	NIR	Prospective cohort study	50 neonates and infants undergoing CHD repair with CPB	Monitoring renal oxygen metabolism in infants after CS allowed for early prediction of AKI.	[63]
	NIR	Prospective cohort study	242 infants (1–12 months) undergoing a VSD repair involving CPB	In infants, intra-operative renal desaturation was associated with an increased risk of AKI following surgical repair of an isolated VSD involving cardiopulmonary bypass. In children undergoing CS, real-time evaluation of renal rSO_2_ using specific levels of change of a 20% reduction for 20 min might be useful in predicting prolonged mechanical ventilation and other adverse outcomes.	[64]
	NIR	Retrospective cohort study	41 patients undergoing CABG with or without CPB	Although non-invasive and continuous tissue oxygenation of the renal region was available, SrtO_2_ could not be used to predict postoperative renal impairment after CABG in adults.	[65]
	NIR	Prospective cohort study	106 children (≤4 years) undergoing CHD repair with CPB	The ability of peripheral tissue deoxygenation to predict postoperative renal impairment suggested that SptO_2_ provided a more accurate indication of the ‘general’ tissue oxygenation status. In children undergoing cardiac surgery, real-time evaluation of renal NIRS using specific levels of change of a 20% reduction for 20 min may be useful in predicting prolonged mechanical ventilation and other adverse outcomes.	[66]
	NIR	Retrospective cohort study	150 adult patients undergoing CS under CPB	In adults undergoing cardiac surgery, regional oxygen saturation in the thigh during CPB was an important marker for predicting postoperative AKI.	[67]
	NIR	Prospective cohort study	87 children undergoing a VSD repair involving CPB	CPB-AKI was strongly predicted by rSO_2_ measured in the thigh during CPB.	[68]
	NIR	Prospective cohort study	48 infants (≤6 months) undergoing CS under CPB	Monitoring intra- and postoperative renal regional saturation in infants may provide an early, non-invasive marker of renal insufficiency following cardiac surgery.	[69]
AKI in preterm infants	NIR	Prospective cohort study	100 premature babies with a gestational age of ≤32 weeks	Low renal rSO_2_ values in the first hours of life in premature babies might play a role in AKI prediction.	[70]
	NIR	Prospective cohort study	35 preterm (<32 weeks gestation) neonates	Preterm neonates at risk of AKI were identified via renal rSO_2_ monitoring. NIRS detected a decrease in renal rSO_2_ prior to changes in serum creatinine and urine output, which was significantly lower in patients with AKI than in those without AKI.	[71]
	NIR	Prospective cohort study	128 preterm (≤32 weeks gestation) infants	Low renal rSO_2_ levels on the first day of life were associated with the development of AKI in preterm infants at 32 weeks of gestation.	[72]
CKD	MIR	Cross-sectional study	52 CKD patients and 6 HCs	ATR-FTIR spectroscopy of salivary urea could provide a viable tool for the rapid and cost-effective diagnosis of stages 3–5 CKD.	[6]
	MIR	Cross-sectional study	14 CKD patients and 14 age- and gender-matched HCs	Thiocyanate (2052 cm^−1^) and phospholipid/carbohydrate (924 cm^−1^) vibrational modes in ATR-FTIR original and second-derivative spectra could be used as salivary biomarkers to distinguish CKD from control subjects.	[73]
DN	NIR	Cross-sectional study	26 DN biopsies and 27 HCs (of whom 25 patients had DM)	Significant correlations were discovered be-tween spectral features and laboratory parameters that indicated glycemic and uremic loads, such as HbA1c, urea, creatinine, eGFR, and proteinuria.	[74]
	MIR	Cross-sectional study	4 NLs, 4 NLDs, and 5 kidneys showing signs of DN	IR imaging can detect critical biochemical changes that occur before morphological changes, allowing for earlier intervention.	[75]
GN	MIR	Cross-sectional study	24 crescentic GN (26–82 years old) and 11 HCs (23–56 years old)	Specific urinary FTIR biomarkers may provide a non-invasive, rapid, sensitive, and novel method for diagnosing inflammatory forms of GN, as well as real-time monitoring of progress and response to treatment.	[5]

### 3.2. Acute Kidney Injury in Very Preterm Infants

AKI is a common complication in preterm newborns. It affects 8–24% of infants receiving care in neonatal intensive care units, with a rate of 18% in infants with very low birth weights [5]. Nephrogenesis is completed in neonates delivered at term, whereas it occurs in premature infants’ extrauterine lives [70,76,77,78,79]. During the transition to extrauterine life, the immature kidney is frequently subjected to acute insults, such as perinatal hypoxia, severe respiratory problems, heart failure, patent ductus arteriosus, birth anemia, and medications. All of these circumstances could result in (subclinical) renal hypoperfusion and hypoxia [70,80,81,82]. Therefore, research on the early indicators of renal damage and hypoperfusion is encouraged [70,76,83]. To detect AKI before irreparable kidney damage occurs in preterm infants, the use of serum creatinine and urine output as diagnostic indicators of AKI is insufficient. This diagnostic weakness makes it difficult to make prompt medical management modifications (such as intravenous fluid administration or nephrotoxic medication adjustments), which could minimize the severity or decrease the duration of AKI. Additionally, this restriction prevents the development of AKI treatments when kidney damage is reversible and treatments may be more successful. Regardless of the precise underlying cause, renal rSO_2_ monitoring may have prognostic value for the development of AKI [71,84,85].

In multiple studies [70,71,72], renal rSO_2_ values in the first postnatal hours appeared to be significantly lower in neonates with AKI than in those without AKI (*p* < 0.05) (Table 2). These results were expanded by a prospective cohort study of 35 preterm neonates (≤32 gestational weeks), showing significant renal desaturation in the first postnatal week (*p* < 0.001) [71]. However, a prospective cohort study of 100 premature infants found that the first 6 postnatal hours were the only time period in which there were significant differences in renal rSO_2_ levels between AKI patients and neonates with normal kidney function (*p* < 0.05). In the first 24 h, these substantial differences disappeared [70]. Additionally, compared to all monitored hours, neonates with AKI spent more hours with renal desaturations <50% (66.6% vs. 28.6%, *p* < 0.001). When comparing the AKI to the no AKI groups, this disparity was true for value cut-offs of 40%, 30%, and 20% (*p* < 0.001) [71].

NIR-detected decreases in renal rSO_2_ levels may serve as an early indicator of AKI onset [70,72]. A prospective cohort investigated the relationship between the development of AKI (serum creatinine >1.5 mg/dL) and early measures of renal perfusion in a cohort of 128 preterm infants [72]. Renal rSO_2_ on the first day of life and resistive index (RI) via renal artery Doppler were two renal perfusion measurements that were significantly lower in patients who developed AKI (*p* < 0.001 and *p* = 0.005, respectively). In addition, logistic regression analysis confirmed this relationship between the development of AKI and renal rSO_2_ and RI (β = −1.3, *p* < 0.001; β = 77.1, *p* = 0.004, respectively). Low renal rSO_2_ values on the first day of life continued to be linked to a high serum creatinine peak from Days 2 to 7 postnatally, even after accounting for potential confounding factors (treated patent ductus arteriosus, caffeine, total plasma protein, mean renal rSO_2_; β = −0.50, *p* = 0.02) [72]. Although it is advantageous to link low renal NIR spectroscopy values on any subsequent day with the onset of AKI, using renal rSO_2_ measurements as a bedside tool to determine immediate care may be more crucial [71].

To distinguish preterm children at risk of AKI from those who are not, the ideal cut-off for renal desaturation was examined. The sensitivity and positive predictive value (PPV) for the diagnosis of AKI using the number of hours spent with renal rSO_2_ < 50% were 64% and 17%, respectively, whereas the specificity and negative predictive value (NPV) were 71% and 95%, respectively. The amount of time spent with renal rSO_2_ values < 20% was associated with the highest relative risk (RR) of AKI (RR: 6.5, *p* < 0.001). Although employing renal NIR spectroscopy to detect AKI appears to have low sensitivity and PPV, the NPV was high. Therefore, if a newborn has normal renal rSO_2_, they may require less frequent serum creatinine monitoring, which could save blood draws and lead to a reduction in the incidence of iatrogenic anemia. The ROC values also imply that the length of time spent below various renal rSO_2_ cut-off values seems to play a key role in identifying individuals who are at risk of developing AKI. For instance, the best ROC values were observed for any 8 or 12 h period with renal rSO_2_ values < 50%, or for any 4 h period with renal rSO_2_ < 40% (AUC = 0.984, *p* = 0.006 and AUC = 0.969, *p* = 0.008, respectively). Future treatment guidelines based on renal rSO_2_ monitoring will need to consider these cut-off values and time frames. To further assess the PPV and NPV for diagnosing preterm newborn AKI, larger investigations are required [71].

A growing body of evidence supports the clinical utility of monitoring renal tissue oxygenation in specific populations at a high risk of immature or abnormal renal function. However, it is unclear whether non-invasive NIRS monitoring of renal tissue oxygenation will have a place in routine clinical practice as serum creatinine measurement remains the gold standard assessment of estimated GFR (eGFR). More research on renal tissue oxygenation in all populations is needed to determine whether there is a link to our current best markers of eGFR, serum creatinine, and cystatin C. Long-term studies are required to correlate neonatal renal tissue oxygenation with childhood and adult kidney function. Significant research is needed to better understand the complex relationship between oxygen delivery and kidney oxygen extraction, as well as concurrent cerebral and systemic oxygenation. Although population-based norms for renal oxygen saturation have been reported, specific thresholds associated with AKI, as well as anticipated temporal and developmental changes, are still unknown. Changes in kidney perfusion may be reflected by changes in renal tissue oxygenation; however, the extent to which these hemodynamic changes affect kidney function may vary. Both renal oxygen saturation and renal oxygen extraction data can help to explain the physiology of acute and chronic kidney injuries. Monitoring renal oxygenation with NIRS is also important for assessing the impact of various therapeutic interventions to preserve kidney function and reduce neonatal AKI [86].

### 3.3. Chronic Kidney Disease

CKD, which affects 8–16% of people globally, is described as a chronic impairment in kidney structure or function (such as a GFR ≤ 60 mL/min/1.73 m^2^ or albuminuria ≥ 30 mg per 24 h) for more than 3 months [6,87,88,89,90,91,92]. The most frequent causes of CKD worldwide are diabetes mellitus and/or arterial hypertension, although other conditions such as GN, infections, and environmental exposures (air pollution, herbal medicines, and pesticides) are also widespread in Asia, sub-Saharan Africa, and many other developing nations [90,93]. CKD risk factors may also arise from genetic predisposition. For instance, the presence of two APOL1 risk alleles and the sickle cell trait, both of which are more prevalent in people with African heritage than in those of European ancestry, may increase the risk of CKD twofold [71,90,94,95,96,97]. However, as mentioned above, traditional serum biomarkers for CKD are insensitive, nonspecific, and increase late in the disease process.

Numerous studies [98,99,100,101] have revealed that in addition to serum creatinine and albuminuria/proteinuria, salivary creatinine, urea, uric acid, cortisol, and phosphate are positively correlated with the severity of CKD and may therefore serve as markers of kidney function. Salivary creatinine and urea levels are considered to be useful clinical indicators for CKD diagnosis with high diagnostic specificity. Since salivary urea correlates favorably with elevated serum urea concentrations in patients with CKD, salivary urea has received special attention and might act as a non-invasive alternative biomarker of CKD [6,102,103]. However, the current clinical use of quantitative salivary urea assays requires expensive and time-consuming commercial kits, which limits the benefit of utilizing saliva and inhibits its wider use as a rapid and simple method for evaluating kidney function [6,104]. When dried in water, pure urea exhibited a noticeable band at 1464 cm^−1^ (attributable to asymmetric C-N stretching vibrations) in the MIR region. However, shifts towards lower wavenumbers were observed when dried in complex mixtures. This is true for the band of urea in dry saliva, which is centered at approximately 1449 cm^−1^ [6,93], most likely due to the retention of water and ensuing variations in the hydrogen-bonding interactions between urea and/or water compared to those of pure dry urea [93,105]. Multiple investigations [6,73] using MIR spectral analysis revealed that salivary urea levels were significantly different between CKD patients and healthy controls (*p* < 0.05). The viability of attenuated total reflection-FTIR (ATR-FTIR) spectroscopy as an alternative approach for evaluating salivary urea concentrations in patients with different CKD stages was examined in a cross-sectional study involving 52 CKD patients and 6 healthy controls. Salivary urea concentrations differed significantly (*p* < 0.05) between the control and CKD stage 3–5 groups (*p* < 0.05). An optimal threshold value of 6.5 mM urea was found, with a sensitivity of 87% and a specificity of 100% (AUC = 0.97). At an ideal threshold of 8.1 mM urea, patients with CKD stages 4 and 5 were distinguished from the control group with 100% sensitivity and 100% specificity (AUC = 1.00), whereas the group of patients with CKD stage 5 on their own had 100% sensitivity and 94% specificity (AUC = 0.95). Salivary urea levels in the same subjects were well linked with serum urea concentrations (r = 0.71, *p* < 0.001) [6]. Other potential salivary biomarkers to distinguish CKD patients from control patients via ATR-FTIR included thiocyanate (SCN^−^, 2063 cm^−1^) and phospholipids/carbohydrates (924 cm^−1^). The best diagnostic threshold value for salivary phospholipids/carbohydrates at 924 cm^−1^ was associated with a sensitivity of 92.9% and a specificity of 71.4% (AUC: 0.88, *p* = 0.0005), while the best model for SCN^−^ at 2063 cm^−1^ showed a sensitivity of 85.7% and a specificity of 71.4% (AUC: 0.76, *p* = 0.016) [73].

The most common treatment for end-stage kidney disease (ESKD) is hemodialysis (HD). Although 150–200 L of dialysate was used for each treatment, there was little continuous monitoring other than measuring pH and conductivity. Continuous monitoring of different substances, particularly in critical patients, can lead to better supervision of dialysis treatment and help to avoid critical situations. Spectroscopic methods are well known for providing access to multiple parameters of interest at the same time [106]. Several studies [107,108,109,110,111,112,113,114] have reported the successful use of MIR or NIR technology to enable the online monitoring of urea (spectral bands at 1630 and 1460 cm^−1^), glucose (spectral bands in the 1200–1000 cm^−1^ region), phosphate (spectral bands in the 1200–1000 cm^−1^ region), lactic acid (the most prominent peak at 1132–1150 cm^−1^), and creatinine (spectral bands at 1720 and 1556 cm^−1^) during dialysis. NIRS monitoring may be a novel method for determining changes in organ oxygenation during HD or the factors influencing tissue oxygenation in patients with CKD [115]. Patients with ESKD who require HD are more likely to develop cognitive impairment and dementia than age-matched controls. Some of the negative outcomes of HD treatment may be explained by a decline in cerebral blood flow (CBF). In a predominantly black patient cohort with 95 prevalent HD patients [116], NIRS was used to assess the cerebral hemodynamic response during dialysis sessions. A novel algorithm, the HD cerebral oxygen demand algorithm (HD-CODA), was developed to automatically detect episodes of cerebral oxygen supply–demand mismatch. The intradialytic mean arterial pressure, heart rate, and volume removal were all associated with this summary measure. However, another study [117] suggested that NIRS underestimated cerebral oxygenation in patients undergoing HD. Compared with magnetic resonance imaging (MRI), positron emission-computed tomography (PET-CT), and transcranial Doppler, NIRS might be a more practical option to assess cerebral oximetry in clinical HD settings because of its ease of portability and lower associated costs [118,119,120]. NIRS could be a proxy for PET to detect intradialytic CBF changes, although both methods capture different physiological brain parameters [121].

Hepato-splanchnic circulation directly influences abdominal organ oxygenation and is important in compensating for the blood volume reduction that occurs in the central circulation during HD with ultrafiltration. In a study [122] of 185 HD patients and 15 healthy volunteers, rSO_2_, a hepatic oxygenation marker that reflects hepato-splanchnic circulation and oxygenation, was measured using an INVOS 5100c oxygen saturation monitor. Hepatic rSO_2_ levels were significantly lower in patients on HD than in healthy controls (56.4 ± 14.9% vs. 76.2 ± 9.6%, *p* < 0.001). BMI, Hb levels, history of cardiovascular disease, mean blood pressure, serum albumin concentration, and colloid osmotic pressure may all influence basal hepatic oxygenation prior to HD. More prospective research is needed to determine whether changes in these parameters, including those experienced during HD, affect hepatosplanchnic circulation and oxygenation in HD patients.

Carbamylation is a significant risk factor for accelerated atherogenesis and mortality in patients undergoing HD. A cross-sectional cohort study [123] of 84 HD patients and 53 healthy volunteers showed that carbamylation, as assessed via NIR analysis of nail proteins, was associated with serum concentrations of uremic toxins and mortality. The second derivative of the peak intensity at 1494 nm, attributed to the N-H amide bands from the NH_2_ of carbamoyl (-CONH_2_) groups, was significantly higher in HD patients than in controls (*p* < 0.0001). More research is needed to determine whether the load of carbamylated nail proteins, as measured via NIR spectroscopy, is a surrogate marker or a hard indicator of mortality risk.

### 3.4. Diabetic Nephropathy

ESKD is mostly caused by DN, which affects 20–40% of individuals with type 1 or type 2 diabetes mellitus (DM) [74,124]. DN is characterized by steady deterioration in kidney function and/or albuminuria [125,126]. High blood pressure and chronic hyperglycemia are the main risk factors for developing DN [127]. However, although not fully understood yet, renal fibrosis, altered renal hemodynamics, oxidative stress, inflammation, hypoxia, and an overactive renin-angiotensin-aldosterone system (RAAS) are significant contributing factors in the pathophysiology of DN [128]. The greatest challenge is determining whether DN is present in the early stages of the disease because histopathological findings in the early stages of the disease are frequently absent [129]. This early detection is of the utmost importance because DN development and progression can be stopped with intensified multifactorial therapies such as RAAS blockade, blood pressure and glucose control, and smoking arrest [128,129].

In a cross-sectional investigation [75], MIR spectral analysis could forecast the onset of DN more accurately. Tissue was collected from four histologically normal kidneys (NL), four histologically normal kidneys from diabetic individuals (NLD), and five kidneys showing signs of DN using human primary kidney biopsies or nephrectomies. In all three glomerular components (glomerular basement membrane, tubular basement membrane, and mesangium), a clear distinction between DN and the NL and NLD cohorts was observed in the 1120–1000 cm^−1^ spectral region. In the glomerular basement membrane, tubular basement membrane, and mesangium of DN biopsies, there were increases in the glycosylation-associated (1030 cm^−1^) and DNA- and glycosylation-associated (1080 cm^−1^) peaks. Importantly, there were remarkable spectral differences in the fingerprint regions of DN patients compared with NLD patients. Therefore, DN, in contrast to the patient’s diabetic status, was most likely the cause of the observed diabetic signature. Furthermore, principal component analysis (PCA) of the 3850–900 cm^−1^ spectral region showed unique clusters in the early biopsies (without clinical or histological DN) that were related to both recurrent and non-recurrent DN in late biopsies. This suggests that it is possible that underlying biochemical alterations have already occurred even when there is no histological proof of DN, and this makes FTIR a desirable tool for DN progression prevention through early medical interventions (Table 2).

Complementary to spectroscopic imaging in the MIR spectral range to identify early biochemical changes prior to histologic changes, NIR spectral analysis could detect post-translational modifications in a straightforward and non-destructive manner [74]. A cross-sectional study compared the NIR spectra of normal renal biopsies (*n* = 27, 22 patients with DM) with those of DN biopsies (*n* = 26). The peak intensities at 1468, 1949, and 2279 nm were considerably lower in patients with DN than in control subjects (*p* = 0.0035, *p* = 0.024, and *p* = 0.0020, respectively), while values at 2082 and 2209 nm were significantly higher (*p* = 0.0058 and *p* = 0.044, respectively) (Table 3). Additionally, all of the control and DN samples were categorized with 100% accuracy using a PCA classification model based on the spectral area between 1700 and 2165 nm. On tissue slices that had not been stained, tests for (de)glycation and carbamylation were carried out to determine the metabolic basis of the most remarkable spectral differences in patients with DN. After carbamylation with a potassium cyanate solution, the peak at 1468 nm, which was mostly attributable to N-H combination bands from the CONH_2_ groups, showed a decrease in intensity. A comparable decline in the peak intensity of the 1468 nm peak was observed after glycation with glucose solution. This peak intensity was barely affected by fructosamine-3-kinase (FN3K) deglycation. Following carbamylation and glycation, the peak at 1949 nm lost some of its intensity. This can be explained by the fact that this region can be attributed to O-H stretching and HOH bending combinations, as well as the N-H combination bands from the CONH_2_ groups. Additionally, FN3K therapy caused the glycated tissue portions to be fully restored, even above baseline levels. It is likely that carbamylation and glycation are key contributors to this spectral discovery because a similar drop was observed in the spectra of patients with DN. Similarly, after the carbamylation and glycation processes, the 2082 nm peak, which was connected to O-H bending and C-O stretching combinations of CONH_2_ groups, displayed a higher intensity. This was corroborated by the use of FN3K, which only partially restored the elevated peak intensity in glycated tissue sections. After glycation and carbamylation, the peak at 2209 nm, which was connected to the C-H and C=O stretching combination bands, showed a noticeable increase in intensity. A slight restoration of the enhanced intensity observed in glycated samples was induced by FN3K therapy. Finally, after carbamylation and glycation, the peak intensity at 2279 nm, associated with the O-H and C-O stretching combinations, exhibited a minor drop, which was also completely restored by FN3K treatment. In the correlation analysis, the strongest associations between peak intensity and HbA1c were observed at wavelengths of 1879, 1987, and 2222 nm (r = 0.46, *p* = 0.014; r = 0.49, *p* = 0.0085; and r = 0.52, *p* = 0.0048, respectively). The glycation experiment, which revealed spectrum alterations in all of these locations and FN3K therapy producing noticeable spectral restorations, confirmed the relationship between these regions and the glycation status. Additionally, both of the intensities at 1403 nm and 1732 nm revealed significant associations with creatinine and eGFR (for 1403 nm: r = −0.44, *p* = 0.012; r = 0.47, *p* = 0.0072; for 1732 nm: r = −0.47, *p* = 0.0084; r = 0.47, *p* = 0.0070, respectively). Based on a machine learning classification model, perfect discrimination was possible in the spectral band between 1700 and 2165 nm, which could be partially linked to both carbamylation (CONH_2_ groups) and glycation (O-H stretching and HOH bending combinations, O-H bending, and C-O stretching combinations) processes. During follow-up, several control patients who were similarly considered to be within the 95% or 90% CI of the DN class model manifested histological or clinical DN-related symptoms. This implies that NIR spectroscopy plays a role in the very early diagnosis of DN, even before histological problems. However, they were also able to distinguish between patients with DM and without DN and those with DN, showing that the biochemical signature does not only reflect a patient’s glycation or carbamylation status. This is significant since not all people with DM and renal illness have DN.

### 3.5. Glomerulonephritis

GN is a heterogeneous group of diseases that clinically presents with a combination of hematuria, proteinuria, arterial hypertension, and reduction in kidney function to a variable degree [130]. GN can be generally classified as immune-complex GN (including infection-related GN, IgA nephropathy, lupus nephritis, and cryoglobulinemic GN), anti-neutrophil cytoplasmic antibodies (ANCA)-associated (pauci-immune) GN, and anti-glomerular basement membrane (GBM) GN [130]. Kidney biopsy with characteristic glomerular inflammation, characterized by increased glomerular cellularity, is currently the gold standard for GN diagnosis [130,131,132].

One study [5] looked for particular FTIR spectrum indicators of kidney damage in the urine of inflammatory GN patients. Among the 24 patients with crescentic GN who were identified as having ANCA-related vasculitis and 11 healthy volunteers, the 1545 cm^−1^ spectral signature was more intense in GN patients than in healthy controls (*p* < 0.05). Additionally, it was clear that patients with moderate-to-severe GN with an eGFR ≥ 60 mL/min/1.73 m^2^ had a higher 1545 cm^−1^ band intensity than those who had an eGFR of less than that. The band at 1545 cm^−1^ was likely attributable to the amide II band of the peptide bonds of the urinary protein. Hence, it is a measure of urinary protein and has the potential to be an earlier and more sensitive marker of kidney injury progression than the currently measured proteinuria (Table 2).

## 4. Conclusions

As demonstrated in the present review, NIR and MIR spectroscopy may have the potential to provide novel screening and diagnostic tools for kidney diseases. Despite the proof-of-concept studies on the potential value of IR spectroscopy to detect AKI and CKD, several sample preparations and, in particular, technology development issues must be addressed to provide a fast, cost-effective method that could replace more traditional clinical screening methods to detect AKI or CKD that require blood samples or other time- and cost-intensive methods. This necessitates the development of simplified, non-specialist spectrometers and/or multiwell plate readers for the simultaneous preparation and analysis of multiple samples [6]. The key benefits of using IR are that, in contrast to many other spectroscopy-based analytical platforms used to analyze urine, such as MS and nuclear magnetic resonance, it can be “manipulation-free”, chemically non-destructive, cost-effective, and possibly less operator-dependent. However, several difficulties remain during clinical translational research and should be taken into consideration in order to solve them. For instance, large-scale randomized control trials and incorporation with current conventional diagnostic approaches are required to prevent bias from small-sample studies and to further validate spectrum biomarkers or signatures for diagnostic or prognostic purposes [5,133,134]. Additionally, with regard to technical issues, standardization (such as sample spectrum collection and pre-analytical processing), the robustness of analytic methodologies, and automation will help to facilitate true clinical translation [5,40,133,135]. Finally, novel studies should be conducted to investigate the changes in NIR or MIR spectra in relation to the progression of kidney disease.

## Figures and Tables

**Table 1:** Overview of the use of the mid-infrared spectrum in the molecular characterization of proteins.

Biomolecule	Spectral Region (cm^−1^)	Functional Group Assignment
Amide A	3310 and 3270 cm^−1^	N-H stretching
Amide I	~1650 cm^−1^	C=O stretching
Amide II	~1550 cm^−1^	C-N stretching N-H in-plane bending
Amide III	1400–1200 cm^−1^	C-N stretching N-H in-plane bending C=O in-plane bending

**Table 3:** Overview of the effect of glycation/carbamylation and fructosamine-3-kinase (FN3K) deglycation on peak intensities at 1468, 1949, 2082, 2209, and 2279 nm [74]. DN: diabetic nephropathy; FN3K: fructosamine-3-kinase, ↓: decrease, ↑: increase. * *p* < 0.05; ** barely restored after FN3K deglycation therapy.

Wavenumber	Functional Group Assignment	Normal vs. DN	Effect of Glycation/Carbamylation	Effect of FN3K Deglycation Therapy
1468 nm	N-H combination bands	↓ *	↓	↑ **
1949 nm	N-H combination bands O-H stretching HOH bending	↓ *	↓	↑
2082 nm	O-H bending C-O stretching	↑ *	↑	↓
2209 nm	C-H stretching C=O combination bands	↑ *	↑	↓

## Data Availability

Not applicable.
